# Editorial der Herausgeber:innen

**DOI:** 10.1007/s12592-021-00404-2

**Published:** 2022-01-10

**Authors:** Fabian Kessl, Karin Bock, Karin Böllert, Rita Braches-Chyrek, Bernd Dollinger, Catrin Heite, Werner Thole, Holger Ziegler

**Affiliations:** 1 Bergische Universität Wuppertal, Wuppertal, Deutschland; 2 Dresden, Deutschland; 3 Münster, Deutschland; 4 Bamberg, Deutschland; 5 Siegen, Deutschland; 6 Zürich, Schweiz; 7 Dortmund, Deutschland; 8 Bielefeld, Deutschland

„Doch von größeren Zusammenhängen, Strukturen oder Machtverhältnissen hatte ich keine Ahnung. Da fehlten mir die einfachsten Erkenntnisse – zum Beispiel die, dass mein Leben auch anders hätte sein können. […] Ich erinnere mich noch genau an den Moment, als ich dachte: Fuck! Wenn meine Eltern woanders gewohnt hätten, hätten wir einen anderen Küchenfußboden gehabt.“ In diesen Worten fasst Resi, die Protagonistin in Anke Stellings Roman *Schäfchen im Trockenen *(Verbrecher-Verlag 2018), den Klassen- und Milieubezug des Wohnens in Gegenwartsgesellschaften in pointierter Form zusammen. Wohnen ist nicht nur zur neuen sozialen Frage geworden, sondern auch zum Manifestationsort von Klassen- und Milieuunterschieden: „Wo kann sich wer das Wohnen (noch) leisten; und wer wohnt wie unter welchen Bedingungen?“ Mit Wohnen verbinden sich aber auch vielfache Fragen des alltäglichen (Über‑)Lebens: Das erfahren alle diejenigen, die von Wohnungslosigkeit betroffen sind, in existenzieller Form schon lange; angesichts eines Wohnungsalltag im Angesicht des anhaltenden Klimawandels erfahren das aber auch andere inzwischen: „Wer kann heiße Sommer im gut isolierten und verschatteten Eigenheim ertragen; wer dagegen ist ihm in der kleinen Dachwohnung oder gar in der Wohnungslosigkeit ausgesetzt?“ Daher ist es kein Zufall, dass Soziale Arbeit und Sozialpädagogik in vielfacher Weise mit Wohnen konfrontiert sind. Umso erstaunlicher ist es, wie wenig das Wohnen bis vor wenigen Jahren Thema war in sozialpädagogischen Debatten und in den Arbeitsfeldern Sozialer Arbeit. Die inzwischen an einigen Stellen entstehenden Diskussionen und Forschungsarbeiten zum Wohnen nimmt der aktuelle „Blickpunkt“ der *Sozialen Passagen* auf und vertieft diese – in fünf einschlägigen Beiträgen zur gegenwärtigen Praxis und Politik des Wohnens. Der Blickpunkt wird von* Christian Reutlinger *(St. Gallen) gemeinsam mit *Fabian Kessl *(Wuppertal) herausgegeben (siehe dazu die „Einführung in den Blickpunkt“).

Im aktuellen „Forum“ diskutieren *Jan Finzi *(Dortmund) und *Janine Kuhnt *(Jena) neue Formen des Wohnens – und nehmen damit losen Anschluss an den „Blickpunkt“ im vorliegenden Heft: Tiny Houses begreifen sie auf Basis einer Analyse der Selbstpräsentationspraxis von beteiligten Akteur:innen als „symbolisierte Selbstverwirklichung“ und zugleich als „Ausdruck von Solidarität mit von Wohnungsnot Betroffenen“. Gerade der letztgenannte Aspekt erweist sich dabei allerdings als höchst voraussetzungsvoll. *Julian Sehmer *(Holzminden) widmet sich in seinem Beitrag der Politik für LGBTI*Q-Personen, die er im Kontext der gesellschaftlichen Auseinandersetzung um die (Be‑)Deutung von Gender und Familie verortet. Die Analyse einer katholischen Predigt des Vorsitzenden der Jugendkommission der Deutschen Bischofskonferenz nutzt *Sehmer*, um zu verdeutlichen, wie sich queerfeindliche Muster mit liberalen Positionen verknüpfen, aber auch rechtspopulistische Deutungsmuster etabliert werden, und welche Konsequenzen das für die Soziale Arbeit hat. Das Selbstverständnis von ehrenamtlichen Jugendhelfer:innen in DDR-Jugendhilfekommissionen steht im Fokus des Interesses von *Diana Düring *(Jena),* Agnes Arp *(Erfurt) und *Birgit Bütow *(Salzburg). Damit rücken sie einen bislang weithin vernachlässigten Aspekt der DDR-Jugendhilfegeschichte auf Basis einer empirischen Vorstudie in den Blick. In dieser widmen sie sich den Motivationslagen der ehrenamtlichen Jugendhelfer:innen und den formal-rechtlichen wie konzeptionell-ideologischen Bedingungen von deren Arbeit. *Philipp Sandermann, Laura Wenzel und Marek Winkel *(alle Lüneburg) befassen sich mit dem aktuellen Forschungsstand zum Thema fluchterfahrener Familien in Deutschland, um auf dieser Basis für die Weiterentwicklung einer heterogenitätssensiblen Sozialen Arbeit zu argumentieren. Ihre Literaturanalyse weist darauf hin, dass in den vorliegenden Forschungsarbeiten vor allem die Besonderung von fluchterfahrenen Familien in den Vordergrund gerückt wird und ordnungs- und sozialpolitisch tradierte Vorstellungen von „geflüchteten Familie“ reproduziert werden. In ihrem Beitrag über die Arbeit in Perspektivklärungsgruppen der stationären Kinder- und Jugendhilfe zeigen *Sascha Dalügge *(Düsseldorf),* Jenni Walther *(Münster), *Johanna Schratz *(Wesseling) und *Nicola Grossheinrich* (Köln), wie die Mitarbeiter:innen die gegenwärtigen Voraussetzungen und den Prozess der Perspektivklärung einschätzen. Der Umgang mit als psychisch und sexuell auffällig geltendem Verhalten der Kinder, die Vernetzung der am Hilfesystem Beteiligten und die Beteiligung der Kinder und Jugendlichen erweisen sich dabei als verbesserungsbedürftig.

In den drei „Forschungsnotizen“ in diesem Heft werden Projekte vorgestellt, die zentrale Fragestellungen für die Soziale Arbeit bearbeiten. So stellen sich *Frank Sowa* (Nürnberg), *Kristian Fahnøe* (Copenhagen), *Alastair Roy, Alan Farrier* (beide Lancashire), *Marco Heinrich* (Nürnberg) und *Mette Kronbæk* (Copenhagen) anhand von drei internationalen Fallstudien die Frage, wie die COVID-19-Pandemie Prozesse der sozialen Inklusion und Exklusion von wohnungslosen Jugendlichen beeinflusst. *Helga Kelle* und *Amanda Edler* (beide Bielefeld) untersuchen, durch welche Fachkräfte, mit welchen Praktiken, Instrumenten und Verfahren sowie in welchen institutionalisierten Netzwerken in Deutschland Risiken, Belastungen und Unterstützungsbedarfe von Kindern im ersten Lebensjahr eingeschätzt werden. Dabei werden insbesondere diejenigen (professionellen) Unter- und Entscheidungen fokussiert, die unterhalb der Eingriffsschwellen bei einer Kindeswohlgefährdung angesiedelt sind. Und schließlich möchte *Veronika Knoche* (Bamberg) mit ihrer Reflexion über forschungsethische, datenschutzrechtliche und forschungspraktische Fragen im Kontext einer ethnografischen Qualifikationsarbeit im Setting Schule, die sich mit der Fragestellung auseinandersetzt, wie Schulsozialarbeiter:innen ihr professionelles Handeln im beruflichen Alltag reflektieren, den Austausch über qualitative Sozialforschung und deren Rahmenbedingungen vorantreiben.

Der Herausgeberkreis und die Redaktion der *Sozialen Passagen* wünschen allen Leser:innen eine ertragreiche Lektüre der vorliegenden Ausgabe.

